# A global comparison of the cost of patented cancer drugs in relation to global differences in wealth

**DOI:** 10.18632/oncotarget.17742

**Published:** 2017-05-09

**Authors:** Daniel A. Goldstein, Jonathon Clark, Yifan Tu, Jie Zhang, Fenqi Fang, Robert Goldstein, Salomon M. Stemmer, Eli Rosenbaum

**Affiliations:** ^1^ Davidoff Cancer Center, Rabin Medical Center, Petach Tikvah, Israel; ^2^ Winship Cancer Institute, Emory University, Atlanta, GA, USA; ^3^ Saint Louis University Hospital, St. Louis, MO, USA; ^4^ First Affiliated Hospital of Dalian Medical University, Dalian, China; ^5^ University College London, London, UK; ^6^ Sackler Faculty of Medicine, Tel Aviv University, Tel Aviv, Israel

**Keywords:** cost, affordability

## Abstract

**Introduction:**

There are major differences in cancer drug prices around the world. However, the patterns of affordability of these drugs are poorly understood. The objective of this study was to compare patterns of affordability of cancer drugs in Australia, China, India, Israel, South Africa, the United Kingdom, and the United States.

**Results:**

Cancer drug prices are highest in the United States. Cancer drugs are the least affordable in India by a large margin. Despite lower prices than in the USA, cancer drugs are less affordable in middle-income countries than in high-income countries.

**Materials and Methods:**

We obtained the prices of a basket of cancer drugs in all 7 countries, and converted the prices to US$ using both foreign exchange rates and purchasing power parity. We assessed international differences in wealth by collecting values for gross domestic product (GDP) per capita in addition to average salaries. We compared patterns of affordability of cancer drugs by dividing the drug prices by the markers of wealth.

**Conclusions:**

Cancer drugs are less affordable in middle-income countries than in high-income countries. Differential pricing may be an acceptable policy to ensure global affordability and access to highly active anti-cancer therapies.

## INTRODUCTION

The cost of cancer drugs is under intense scrutiny. Drug prices at market launch in the United States have increased significantly in recent years [1], however prices alone should not be considered in isolation from clinical benefit. Perhaps more important is the concept of value which can be measured using cost-effectiveness analyses. While the value of some cancer drugs is high, recent studies have demonstrated that some cancer drugs provide low value [2, 3]. Value frameworks have also been developed by professional cancer societies to provide guidance to patients, physicians and policy-makers [4, 5].

While the cost and value of cancer drugs have recently gained considerable attention, an additional factor of economic importance must be considered – namely affordability. The high cost of cancer drugs places a financial burden on both society as well as patients and their families. In the United States, individuals diagnosed with cancer are 2.7 times more likely to declare bankruptcy, than individuals without cancer [6]. Cost, value and affordability of cancer drugs vary around the world. While the clinical benefit of a cancer drug is usually similar around the world, other economic factors may vary significantly. Drug prices are known to be the highest in the United States with lower prices around the world [7, 8]. There is wide variation in both national and personal wealth around the world, thus impacting affordability of cancer drugs.

Cancer care is undergoing a revolution with the arrival of new therapies such as immunotherapy, monoclonal antibodies, and targeted therapy. In 2015 alone, 22 new drugs or indications gained approval in the United States by the Food and Drug Administration [9]. But economic issues must be considered related to these therapies. What is the value of these therapies? How affordable are these therapies for cancer patients around the world?.

The aim of this study was to systematically measure the differences in both prices of cancer drugs and wealth levels between high-income countries (Australia, Israel, the United Kingdom, and the United States) upper-middle-income Countries (China and South Africa) and lower-middle-income countries (India). We analysed drug prices and national wealth in all 7 countries and used these to make inferences about affordability.

## MATERIALS AND METHODS

### Drugs selection and doses

We created a list of all cancer drugs that gained FDA approval since 1995 [9]. Based on the first FDA approval, we calculated the total drug dose required for four weeks of treatment. We used a weight of 82 kg and a body surface area of 1.86 m^2^ based on average height and weight in the US, as used in a prior study [2]. We searched for the drug prices in Australia, China, India, Israel, South Africa, United Kingdom, and United States. We selected these countries based on both local knowledge and some level of price availability. There were many prices that were unavailable in all countries. We therefore reduced the basket of drugs to include only those that were available in all seven countries. We removed drugs if the patent had expired at the time of analysis.

### Countries and price sources

We collected drug prices between November 2015 and January 2016. We aimed to capture both retail prices as well as discounted prices. In many countries there are several different prices for drugs, given the opportunity for discounts and rebates based on negotiations between individual payers and vendors. However there is a significant lack of transparency regarding such prices, potentially with varying degrees of discounting between countries and within countries. In several countries, it was not possible to obtain the discounted prices, but only the retail prices. In order to make comparisons that we felt to be as valid as possible, we chose to only analyse retail prices, given their availability worldwide. When we found multiple price options for the same drug, we selected the lowest price.

To capture the retail prices, we used the following sources. For Australia, we used the pharmaceutical benefits scheme [10]. For China, we used the Chinese Government Pharmacy Information Website [11] in addition to data from the pharmacy department of The First Hospital affiliated with Da Lian Medical College. For India, we used Medline India and drugsupdate.com [12, 13]. For Israel, we used the prices from the Israel Ministry of Health [14]. For South Africa, we used the medicine price registry [15]. For the United Kingdom, we used the British National Formulary [16]. For the USA, we used the Average Wholesale Price [17].

### Currency conversion

We converted prices from local currencies into US dollars using 2 methods:

1. Foreign Exchange rates – We used the foreign exchange rates (FOREX) on January 19th 2016 to perform the conversion [18].

2. Purchasing Power Parity – We used Purchasing Power Parity (PPP) rates provided by the World Bank to perform the conversion. [19] Rates for PPP are estimated based on the cost of purchasing a similar basket of goods in different countries. Using PPP is advantageous as values do not fluctuate significantly with time, which may be the case for foreign exchange rates [20].

### Wealth estimation

We estimated wealth within each country using 2 surrogate markers:

1. GDP per capita - we used the Monthly GDP per Capita at Purchasing Power Parity, provided by the International Monetary Fund (IMF) [21] (Supplementary Figure 2).

2. Average salary – Average salaries per month were obtained from The Statistics Portal, which uses data from the International Labour Organization (Supplementary Figure 3) [22].

### Affordability estimation

To understand affordability in each country, we divided the drug prices by the markers of wealth. The purpose of this calculation was not to estimate how much money was actually spent by individual people or payers, or whether such a percentage was either inappropriate or appropriate. Rather, the calculation was simply to enable comparisons in patterns of affordability between countries.

## RESULTS

### Drugs and doses included

We found 99 cancer drugs approved by the FDA since 1995. Following application of exclusion criteria as described in the methods, this list was reduced to 8 patented cancer drugs (bevacizumab, bortezomib, dasatanib, erlotinib, imatinib, pemetrexed, rituximab, and trastuzumab). The drugs and doses included in the analysis are listed in Table 1.

**Table 1:** Drugs, doses and retail prices

Drug	4 weekly dose (mg)	4 Weekly Retail Price (US$)													
		India		China		South Africa		Israel		UK		Australia		USA	
		XR	PPP	XR	PPP	XR	PPP	XR	PPP	XR	PPP	XR	PPP	XR	PPP
bevacizumab	820	4,291	19,006	2,325	4,364	2,035	6,850	2,603	2,620	2,668	2,714	573	543	6,827	6,827
bortezomib	9.672	917	4,061	2,224	4,174	895	3,013	2,786	2,803	2,966	3,017	4,040	3,824	5,339	5,339
dasatinib	2800	2,773	12,282	693	1,301	1,292	4,349	3,528	3,551	3,527	3,587	3,266	3,091	11,599	11,599
erlotinib	4200	418	1,853	2,796	5,248	1,380	4,646	2,458	2,474	2,368	2,408	1,204	1,140	9,964	9,964
imatinib	11200	146	649	3,549	6,661	575	1,936	2,365	2,380	2,414	2,455	2,466	2,334	11,336	11,336
pemetrexed	1240	458	2,028	3,672	6,891	2,217	7,463	3,992	3,011	2,794	2,842	2,770	2,622	9,042	9,042
rituximab	930	2,112	9,356	6,022	11,302	2,076	6,990	2,366	2,381	2,290	2,329	2,711	2,566	8,346	8,346
trastuzumab	656	2,761	12,228	7,599	14,261	2,397	8,068	1,798	1,809	2,507	2,550	3,302	3,125	6,849	6,849

XR = price in US$ when converted from local currency using foreign exchange rates.

PPP = price in US$ when converted from local currency using purchasing power parity.

### Global differences in drug prices

**Figure 1:**
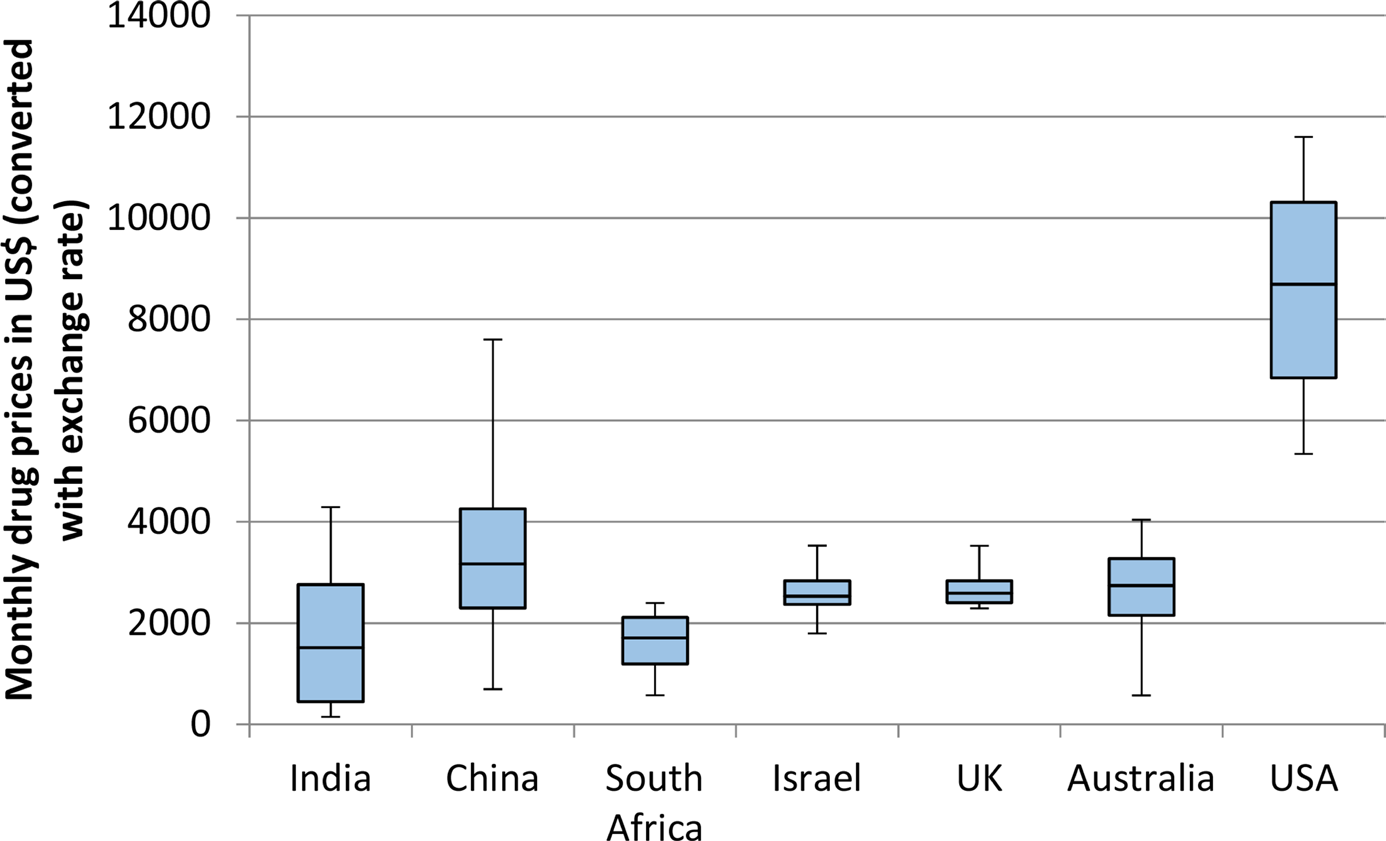
Monthly price of 8 patented cancer drugs in 7 countries Prices were converted from local currency to US$ using exchange rates on 19th January 2016. For each country, the horizontal line represents the median, the box represents the lower and upper quartiles, and the whiskers represent the lowest and highest values.

**Figure 2:**
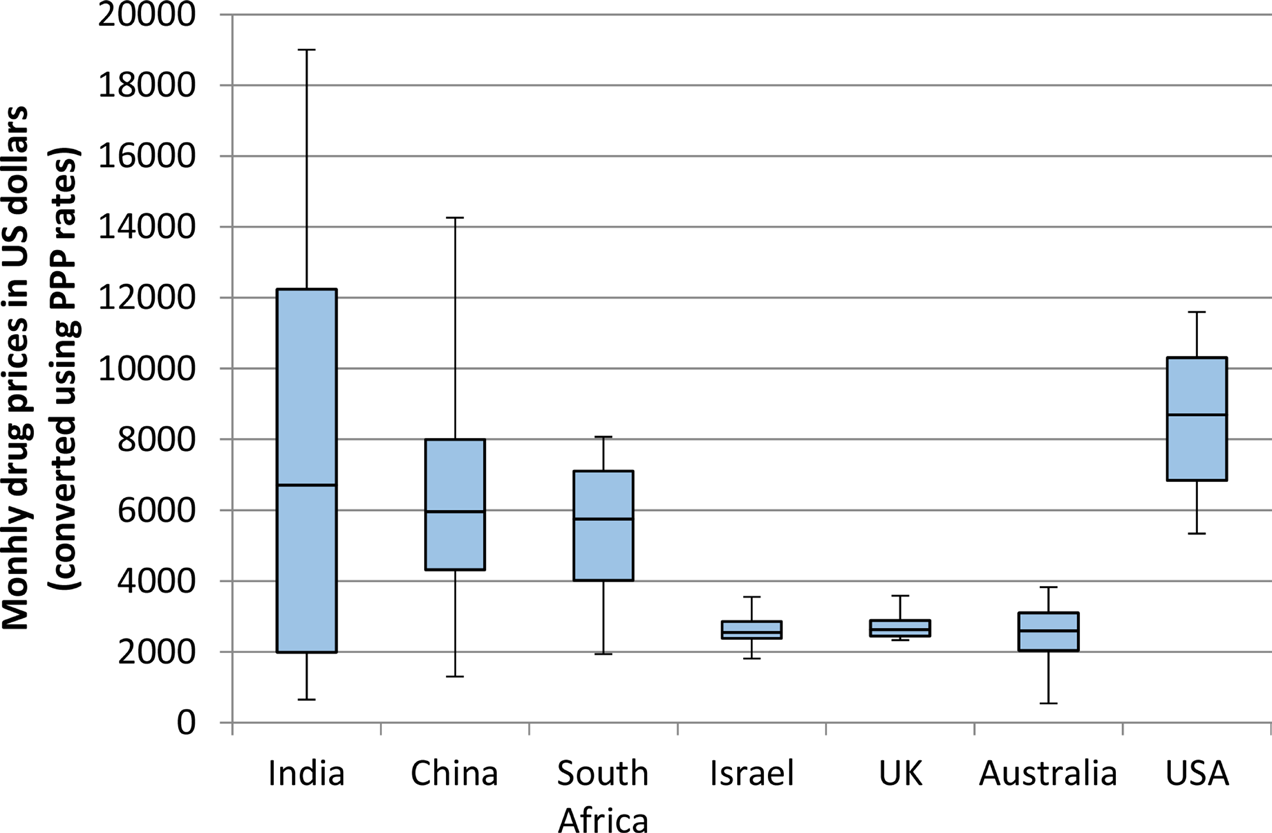
Monthly price of 8 patented cancer drugs in 7 countries Prices were converted from local currency to US$ using purchasing power parity. For each country, the horizontal line represents the median, the box represents the lower and upper quartiles, and the whiskers represent the lowest and highest values.

The differences in drug prices were highly dependent on the method used to convert local currencies into US dollars. When using foreign exchange rates, the median drug price was highest in the USA ($8694) and for the remaining 6 countries, the range of median prices was from $3173 to $1515. When using purchasing power parity to convert from local currency to US$, the pattern of prices was different. The USA median price remained $8694 as the base currency was US$, and this remained the highest price globally. The median prices in all high-income countries remained similar to those using foreign exchange rates. However the median prices in India ($6709), China ($5954) and South Africa ($5748) were significantly higher than those using foreign exchange rates. All drug prices are demonstrated in Table 1 and Figures 1 and 2.

### Global differences in wealth

Based on classification by the World Bank related to Gross National Income, Australia, Israel, the United States and the United Kingdom are High Income Countries. South Africa and China are Upper Middle Income Countries. India is a Lower Middle Income Country. Supplementary Table 1 details these classifications. Rates for GDP per capita and average salaries are demonstrated in Supplementary Figures 2 and 3 respectively.

### Global differences in affordability patterns

**Figure 3:**
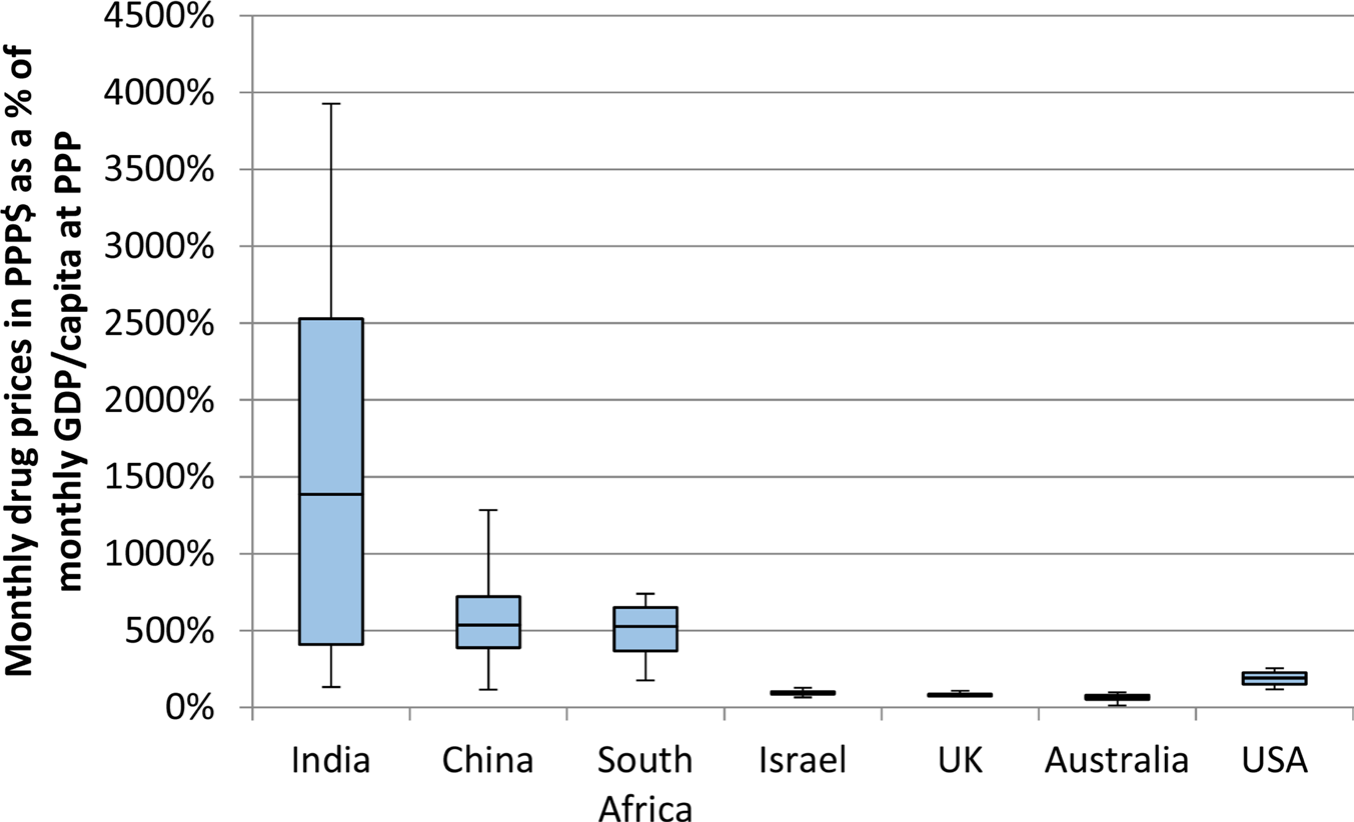
Comparable affordability of 8 patented cancer drugs in 7 countries The monthly prices in PPP$ of 8 drugs (from Figure 2) were divided by the monthly GDP per capita at purchasing power parity.

**Figure 4:**
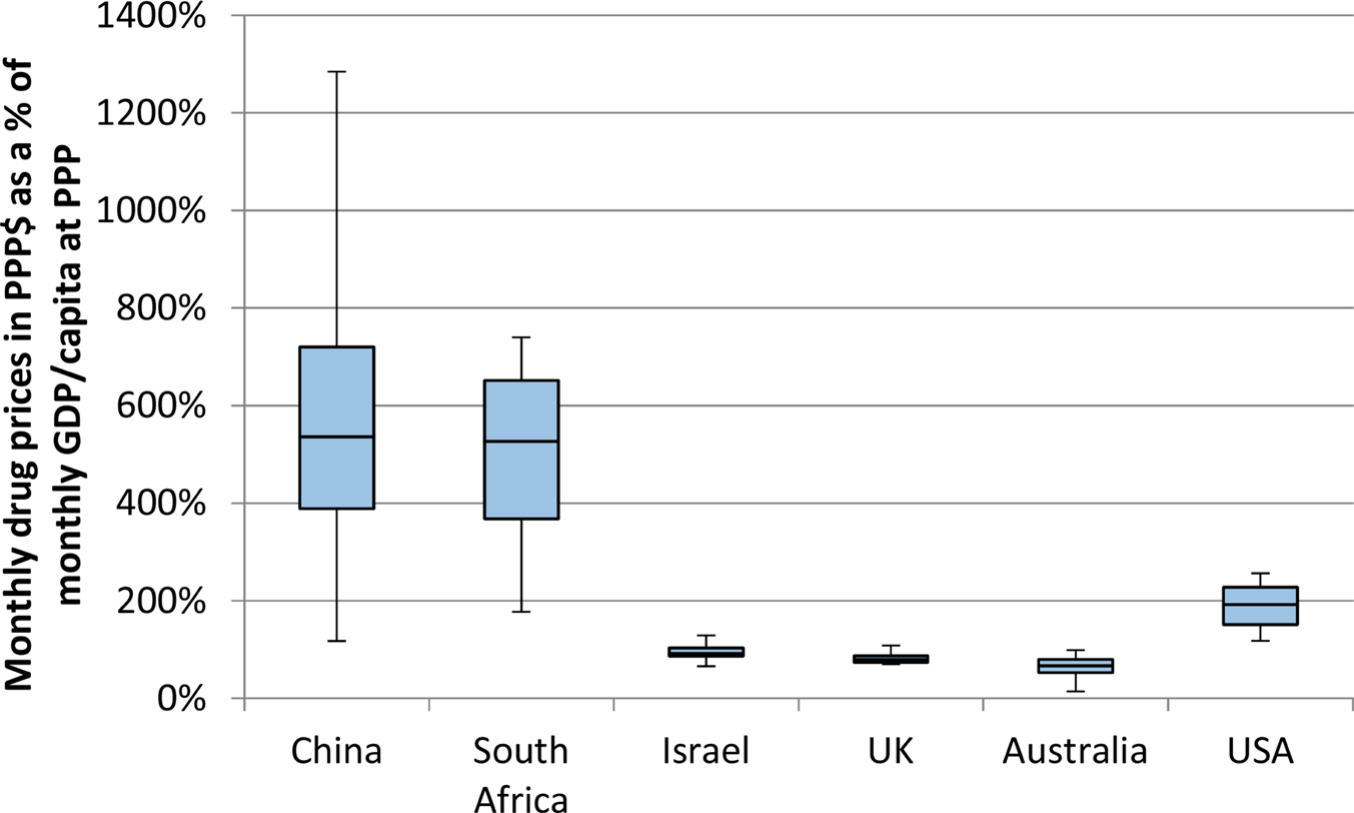
This Figure uses the same data as Figure 3, but with the exclusion of India, in order to see more closely the differences between the remaining 6 countries

There were major differences in patterns of affordability between countries. Drugs were significantly less affordable in India than in other countries by a vast margin. Furthermore, drugs in China and South Africa were less affordable than in all high-income countries, including the US where prices were considerably higher. These differences were driven by lower levels of wealth in middle-income countries. The patterns in Figures 3 and 4 demonstrate the difference using GDP per capita as a marker for wealth. The pattern of affordability was identical when using average salary as a marker for wealth. (Supplementary Figure 1)

## DISCUSSION

In this study we have demonstrated that there are major differences in both affordability patterns and prices of patented cancer drugs around the world. Drug prices are highest in the USA, however drugs appear more affordable in other high-income countries (Israel, UK and Australia) than in the USA and middle-income countries (China, India and South Africa). Although prior studies have demonstrated price differences around the world [7, 8] our study was novel in that it incorporated a range of price differences across countries with significant variability in wealth. To our knowledge, this study was the first of its kind to link the price of cancer drugs to affordability using international markers of wealth.

There is a clear difference in drug prices depending on whether prices are converted from local currency to US dollars using exchange rates, or purchasing power parity. The reason for this difference is driven by the difference in conversion rates. While the foreign exchange rates and purchasing power parity rates are similar for high-income countries they are considerably different for middle-income countries. For example, there are 0.71 UK pounds to one US dollar using FOREX, and there are 0.70 UK pounds to one US dollar using PPP. However there are 66.92 Indian rupees to one US dollar using FOREX, but only 15.11 rupees to one US dollar using PPP. Using PPP to convert drug prices to US$ provides interesting results. We see that although prices may appear somewhat comparable between countries using foreign exchange rates, when we incorporate purchasing power parity, there are significant differences. These differences essentially relate to the difference in the value of US currency between countries.

In understanding differences in wealth between countries there may be some debate regarding the most appropriate metric to use, as GDP per capita does not incorporate personal income that may be impacted by unemployment levels, retirement age, and social patterns of employment. By comparing affordability using both GDP per capita and average salary we have demonstrated that both metrics lead to the same patterns of results for affordability. Using these metrics we have demonstrated large differences in levels of affordability around the world, with drugs being the least affordable in India.

There are limitations in our study. There was some selection bias in our basket of drugs. We started with all cancer drugs approved by the FDA since 1995. However we reduced the list to only the drugs for which we were able to find prices in all countries. By this selection process we ultimately excluded from the analysis some drugs that may have been inaccessible from the outset, perhaps due to a low level of affordability. Nevertheless, the selected 8 drugs represent monoclonal antibodies, targeted agents, as well as cytotoxic chemotherapy. We speculate that some drug prices may not be available due to the drug not being publicly marketed in certain countries. We used a weight of 82 kg to calculate doses, which may be inappropriate in different countries. Using monthly costs may be less appropriate to using total treatment costs. The nonrandomized selection of countries in our analysis limits our ability to extrapolate these data to the whole world. We used retail prices from around the world. As previously described, the transparency in drug pricing around the world is a major challenge. In many countries it is impossible to know the actual net amount of money that changes hands following discounts and rebates. Given the greater availability of retail prices, these were used, however it is possible that the level of discounting varies between countries and within countries. In addition, wholesale distribution costs may vary around the world, which may also differentially impact the final costs around the world. Precise affordability is challenging to compare between countries as there is variability as to whether drugs are publicly reimbursed, or the cost falls on individual patients and their families. Additional patient assistance programs, such as the Glivec International Patient Assistance Program (GIPAP), were not incorporated into this analysis [23]. In understanding the levels of wealth between countries we used gross international estimates such as GDP per capita and average salary. However these metrics do not account for varying levels of wealth inequality within countries. Our initial study could be followed up with a more in depth study, which could attempt to account for some of these limitations. For such a study, increased transparency of actual drug prices around the world would be essential.

While the economics of cancer care have gained considerable attention in recent years, the underlying economic challenges are not new. 15 years ago, similar questions were being asked related to HIV care. The cost of highly active anti-retroviral therapy (HAART) was high but this was coupled with a high level of clinical benefit. The major challenge was affordability. While patients in western countries began to live longer, the drugs were out of reach for most of sub-saharan Africa due to lack of affordability. Ethical, legal and political battles ensued, and ultimately drug prices were reduced, thus improving global affordability, access and survival.

Two important questions arise from this analysis. Firstly, is the drug market different from other markets in terms of price differences? In most markets the prices for identical products varies around world. This phenomenon may be related to supply and demand in addition to differences in costs related to production, transportation, and labour. However, one may argue that social, political and ethical consideration should be given to identifying whether a product is a luxury or essential item, in order to justify pricing. The second question is both philosophical and political. Should identical drugs have identical prices around the world irrespective of where they are purchased, or should they be different? If they are different, should prices simply be based on market forces or should they be related to wealth in order to provide equivalent levels of affordability worldwide?.

The answers to these questions are by no means simple. However, in attempting to answer these questions there is an important factor that must be considered – efficacy and value. We must make a clear differentiation between highly active anti-cancer therapies (HAACT) and those that are not highly active. The drugs included in our analysis included drugs with varying levels of anti-cancer activity. Bevacizumab increases overall survival by approximately 6 weeks in patients with metastatic colorectal cancer [24], while imatinib has converted chronic myelogenous leukaemia, once a fatal disease, into a disease with long term survival for the majority of patients [25]. Furthermore trastuzumab has greatly increased the potential for cure in patients with *HER2* positive localized breast cancer [26]. There may be differences in outcomes between countries following treatment with the same drug. One may consider that attention to prices and affordability in poor countries should be devoted only to HAACT. The American Society of Clinical Oncology considers a clinically meaningful survival benefit to be 3–6 months [27]. Perhaps HAACT in middle-income countries should comprise only the agents with a potential for cure or to extend overall survival by a large magnitude, decided by the individual societies. Further consideration should be given to creating a flexible threshold, which could potentially be used formally by the World Health Organisation in updating the list of essential medicines. Policy attention could then be focussed upon ensuring global affordability of HAACT, as was the case 15 years ago for HAART.

Differential pricing may be problematic for manufacturers due to the concerns of cross-country importing leading to loss of revenue. Nevertheless, a recent example of differential pricing has been supported by drug manufacturers. New drugs for hepatitis C have been widely criticized due to their high price, despite the excellent clinical benefits [28]. However there is significant differential pricing around the world for these drugs [29]. Industry and government partnerships placed restrictions on sales with close monitoring of drug compliance and usage in order to prevent the development of a black market.

Major challenges are expected in the years ahead to pay for the multitude of cancer drugs that have recently been developed, at an increasing financial cost to public and private payers around the world. In some ways this may be reminiscent of the challenges faced in the HIV field 15 years ago. At that time there were large numbers of people worldwide who were unable to gain access to HAART due to drug prices that were unaffordable outside of high-income countries [30]. The health outcomes were devastating, and public protests combined with additional lobbying led to a reduction in prices, ultimately improving access and outcomes for these patients. The case of cancer is both similar and different. Inaccessible cancer drugs with only a minimal level of efficacy may not justify significant efforts to reduce prices. Conversely, highly active anti-cancer therapies that are inaccessible due to price may justify aggressive price negotiations in order to improve access. Drug costs are only a small fraction of the total cost of cancer care. To improve affordability of cancer care, attention must be focused not only on drug costs but also on costs of end-of-life care, diagnostics, surgery and radiation. One of the greatest future challenges in cancer medicine and policy will be to ensure global access to highly active anti-cancer therapies, while maintaining incentives for future research to improve the outcomes for cancer patients around the world.

## SUPPLEMENTARY MATERIALS FIGURES AND TABLES


