# Supplementary material for: A global comparison of the cost of patented cancer drugs in relation to global differences in wealth
Source: Oncotarget. 2017 May 9;8(42):71548–55. doi: 10.18632/oncotarget.17742 (PMC5641070; doi:10.18632/oncotarget.17742)
Supplement: Supplementary file 1 [file oncotarget-08-71548-s001.pdf]

A global comparison of the cost of patented cancer drugs in relation to global differences in wealth

Supplementary Materials

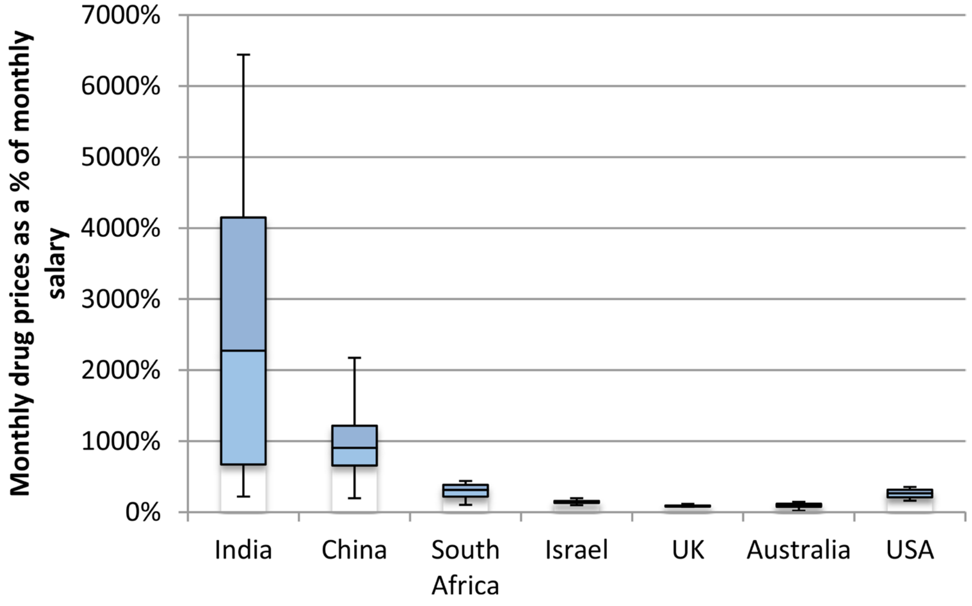

Supplementary Figure 1: Box and whisker chart demonstrating the comparable affordability of 8 patented cancer drugs in 7 countries. The monthly prices in PPP$ of 8 drugs (from Figure 2) were divided by the monthly average salary at purchasing power parity.

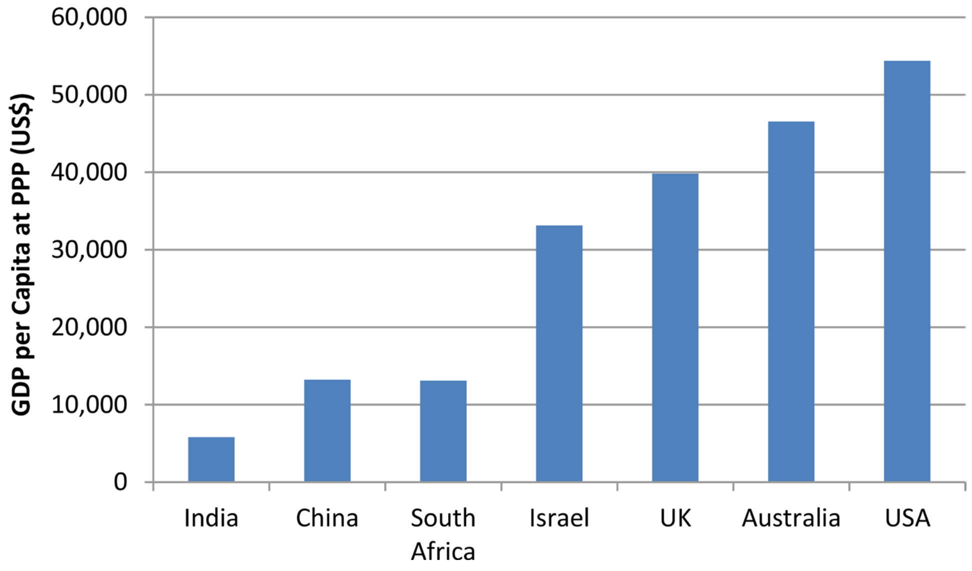

Supplementary Figure 2: GDP per capita rates as provided by the IMF.

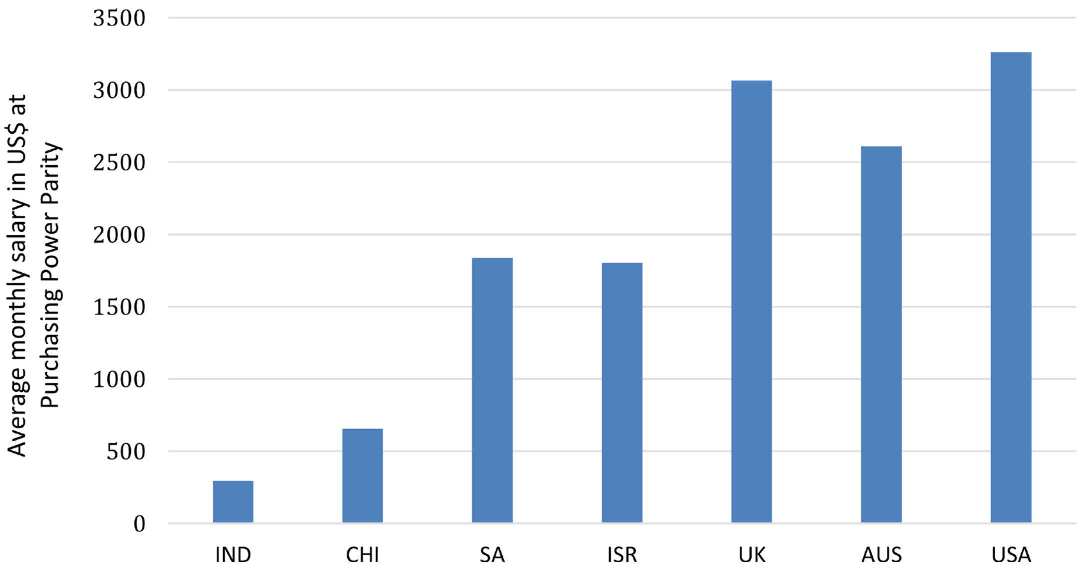

Supplementary Figure 3: Average monthly salary in US$ at Purchasing Power Parity

Key Points

Question: What are the differences in affordability of cancer drugs in Australia, China, India, Israel, South Africa, the United Kingdom, and the United States.

Findings

Cancer drugs are the least affordable in India by a large margin. Despite lower prices than in the USA, cancer drugs are less affordable in middle-income countries than in high-income countries.

Meaning

Differential pricing may be an acceptable policy to ensure global affordability and access to highly active anti-cancer therapies.

Supplementary Table 1: Classification of income level of country as defined by world bank based on GNI per capita

Description	Gross National Income (GNI) per capita	Countries
Lower Middle Income (LMIC)	$1,026–$4,035	India
Upper Middle Income (UMIC)	$4,036–$12,475	China, South Africa
High Income (HIC)	> $12,476	Australia, Israel, UK, USA

Source: https://datahelpdesk.worldbank.org/knowledgebase/articles/906519.

Supplementary Table 2: Average life expectancy at birth

Country	Life Expectancy (years)
Australia	82.8
China	76.1
India	68.3
Israel	82.5
South Africa	62.9
UK	81.2
USA	79.3

Source: World Health Organization (2015).
http://gamapserver.who.int/gho/interactive_charts/mbd/life_expectancy/atlas.html.
